# Efficacy of copper nanoparticles encapsulated in soya lecithin liposomes in treating breast cancer cells (*MCF-7*) in vitro

**DOI:** 10.1038/s41598-023-42514-2

**Published:** 2023-09-20

**Authors:** Shaimaa A. Ahmed, Mohamed H. Gaber, Aida A. Salama, Said A. Ali

**Affiliations:** 1 Biophysics Branch, Physics Department, Faculty of Science, Al-Azhar University (Girl’s Branch), Cairo, Egypt; 2 Biophysics Department, Faculty of Science, Cairo University, Giza, 12613 Egypt

**Keywords:** Biophysics, Biotechnology, Cancer

## Abstract

Cancer is one of the leading causes of death, which has attracted the attention of the scientific world to the search for efficient methods for treatment. With the great development and regeneration of nanotechnology over the last 25 years, various nanoparticles in different structures, shapes and composites provide good potential for cancer therapy. There are several drugs approved by FDA used in breast cancer treatment like Cyclophosphamide, Doxorubicin Hydrochloride, Femara, Herceptin, etc. Each has several side effects as well as treatment, which limits the use of drugs due to heart failure, pulmonary dysfunction, or immunodeficiency. Recently, such side effects are greatly reduced by using innovative delivery techniques. Some drugs have been approved for use in cancer treatment under the concept of drug delivery, such as Doxil (liposomal loaded doxorubicin). The purpose of this study is to investigate the effect of copper nanoparticles (CuNPs) as a drug model for cancer treatment, either in their free form or encapsulated in Soy lecithin liposomes (SLP) from plant origin as a cheap source of lipids. CuNPs were prepared by the chemical reduction method and loaded onto SLP through the thin film hydration method. The drug model Cu/SLP was successfully combined. The characteristics of the free CuNPs, liposomes, and the combined form, zeta potential, size distribution, drug encapsulation efficiency (EE%), drug release profile, Fourier transform infrared (FTIR), and transmission electron microscopy (TEM), were checked, followed by an in vitro study on the breast cancer cell line *Mcf-7* as a model for cytotoxicity evaluation. The optimal Cu/SLP had a particle mean size of 81.59 ± 14.93 nm, a negative zeta potential of − 50.7 ± 4.34 mV, loaded CuNPs showed an EE% of 78.9%, a drug release profile for about 50% of the drug was released after 6 h, and FTIR analysis was recorded. The cytotoxicity assay showed that the IC_50_ of Cu/SLP is smaller than that of free CuNPs. These results give clear evidence of the efficacy of using the combined Cu/SLP rather than CuNPs alone as a model drug carrier prepared from plant origin against cancer, both medically and economically.

## Introduction

Breast cancer is the most diagnosed cancer in women, representing approximately 24.7% of all cancers worldwide, with a mortality rate of 15.5%^1,2^. The high-incidence areas are Western Europe and the United States, whereas the low-incidence areas are Asia and Africa. There are different factors that are considered risk factors for developing breast cancer, such as family history, gene mutation, especially BRCA1 and BRCA2, lifestyle and high-fat diet, and environmental factors such as exposure to ionizing radiation, smoking, and alcohol^3^. Chemotherapy is the best choice for breast cancer treatment before or after surgery if needed. Doxorubicin is the main drug used in breast cancer therapy. It is an antibiotic drug, an oxidized unstable metabolite, used separately or mixed with other types of chemotherapy, such as cisplatin and vincristine. The main drawbacks of Dox are its high cost and high toxicity; they can cause cardio-and heart failure and affect cancer cells as well as highly active normal cells in the body, such as the liver, brain, and kidney^4^. The main aim of this research was to find another drug for breast cancer therapy (CuNPs) and investigate its effect as a drug model either in its free form or encapsulated in soy lecithin liposomes (SLP) of plant origin as a cheap source of lipids.

Nanoparticles (NPs) are a class of material whose size is reduced to the nanometer length scale, which greatly affects its electronic and thus chemical properties. These unique properties provide great potential for the nanoparticles to be used in many applications, such as biomaterials applications, cosmetics, electronics, magnetics, pharmaceutical manufacturing, food preservation, water treatment, and drug delivery applications^5^. There are two categories of nanoparticles: (a) organic nanoparticles (such as liposomes, polymers, and micelles) and (b) inorganic nanoparticles (such as iron oxide, CuNPs, gold, and silica). Organic nanoparticles have a broad range of applications, such as vaccination, long-lasting delivery systems, hemostasis, fungal and parasitic treatment, and anesthesia. Inorganic nanoparticles have a variety of applications such as imaging, anemia treatment, cancer treatment, and thermal ablation^6,7^. Copper is an element essential for metabolic processes in animals and humans^8^. It is needed for cross-linking of connective tissues as well as lipid and iron metabolism, and multiple physiological processes like mitochondrial respiration, metabolism of energy, and antioxidation; however, high concentrations induce toxicity^9,10^. Because of their low prices and availability, copper nanoparticles (CuNPs) are used in several industries, especially medical and biological applications. The physicochemical characteristics of CuNPs, such as chemical composition, charge modulation of reactive oxygen species (ROS), size, and surface region, help in medical applications^11^.

Copper nanoparticles are highly conductive metals compared to other metal nanoparticles^12^. In 2020, oxidative stress generated by the development of extracellular ROS in *MCF-7 cells* treated with CNPs was shown to degrade membrane lipids, resulting in cell shrinkage and death. Fragmentation causes cell death. According to these findings, copper nanoparticles could be promising candidates for the development of anticancer drugs^13^. CuNPs are popular owing to their optical, catalytic, mechanical, and electrical properties; they are more highly conductive and cost-effective than gold and silver; they have excellent electrical conductivity, strong near-infrared absorption, and significant photothermal capabilities; and they have been widely used in photothermal therapy and cancer photoimaging. They have a large specific surface area and can be used to load several anticancer medicines. When exposed to light, they generate a significant number of reactive oxygen species (ROS), which can be used in photodynamic therapy^14,15^. In patients with cancer or any malignancy, it has been demonstrated that they have a much higher blood copper level. Therefore, the equilibrium copper level plays an important role in cancer cell survival^16^. The regulated death of human cells that depends on copper concentration differs from the known death mechanism because it is dependent on mitochondrial respiration through the direct binding of copper to lipoylated products of the tri-carboxylic acid cycle. This binding leads to protein aggregation, and loss of iron-sulfur cluster protein, followed by proteotoxic stress, leads to cell death^17^.

The phospholipids were found in all living systems, whatever their plant or animal origin. In plants of origin, soybean is the major source of phospholipids. Lecithin is a commercial type of phospholipid extracted from soybeans called soy lecithin. Soy lecithin liposomes (SLP) are a type of nanocarrier from the first generation. They are made of a complex mixture of phospholipids with a hydrophobic tail and a polar head. When put in a solution, these phospholipids self-assemble to form sphere rings the size of nanometers. Liposomes can be used to deliver both hydrophobic and hydrophilic drugs^18,19^. Liposomes can entrap a wide range of compounds, including antibiotics, proteins, colors, peptides, antioxidants, nucleic acids, enzymes, and medicines. These vesicles are biocompatible with the human body and are easily absorbed by cells^20^. In recent years, several phospholipid-related formulations, including Doxil®, Cleviprex®, Valium®, and Silybin PhytosomeTM, have been used in clinical trials with positive results^21^. These SLP are more economical, safer, stable, available in both purified and non-purified forms, and less costly for laboratory and pharmaceutical production of liposomes^22^. In this study, copper nanoparticles and Soy lecithin liposomes (Cu/SLP) were prepared through three steps that included chemical preparation of CuNPs, liposomal preparation through the thin film hydration method, and finally conjugation of CuNPs with liposomes. The purpose of this study is to investigate the effect of copper nanoparticles (CuNPs) as a drug model for cancer treatment, either in their free form or encapsulated in Soy lecithin liposomes (SLP) from plant origin as a cheap source of lipids. The physical and chemical properties of Cu/SLP were characterized by size distribution, zeta potential, transmission electron microscope (TEM), molecular structure by using Fourier transform infrared (FTIR), drug loading efficiency, drug release by using a dialysis bag, and in vitro cell line toxicity by using *Mcf-7*.

## Materials and methods

Soy lecithin powder (C_35_H_66_NO_7_P) was purchased from bulk-supplemtnts.com, 7511 Eastgate Rd., Henderson, NV 89011, USA, with a molecular weight (MW) of 643.87 gm/mol. Cholesterol (C_27_H_46_O) with MW of 386.65 gm/mol, purchased from Advent Chembio Privat Ltd. Chloroform (CHCl_3_), with a MW of 119 gm/mol. Ethanol (C_2_H_5_OH) has a concentration of 99%, a melting point of − 114.1 °C, and a MW of 46.07 gm/mol. Copper (II) sulfate pentahydrate (CuSO_4_.5H_2_O), a bright blue crystal, has a MW of 159.609 gm/mol. Ascorbic acid, or Vitamin C (C_6_H_8_O_6_), is a water-soluble vitamin with a MW of 176.12 gm/mol. Sodium Hydroxide (NaOH) with MW: 39.997 gm/mol and potato starch (C_6_H_10_O_5_)n, white powder, all purchased from Alfa chemical stores. Sodium chloride (NaCl) as saline with pH 5.5, a MW of 58.44 gm/mol, deionized water (DI water), and a dialysis bag made from regenerated cellulose (RC) with glycerol as a protector from embrittlement, with a pH range of 2–12 and a temperature of 4–60 °C, with a diameter of 21 mm and a length of 5 m with a molecular weight cutoff of 5 KDa (about 6 cm of the dialysis tubing bag used in our experiment).

### Preparation of Cu nanoparticles (CuNPs)

Chemical reduction, sonochemical reduction, microemulsion techniques, electrochemical, hydrothermal, sol–gel synthesis, polyol processes, biological synthesis, and microwave-aided techniques are the principal chemical methods for creating copper nanoparticles. Laser ablation, vapor-phase synthesis, mechanical milling, and pulsed wire discharge are all physical methods for producing copper nanoparticles^23^. CuNPs were prepared using the chemical reduction method of Khan et al.^24^ with some modifications. This method is preferred because of its low cost, ease of operation, rapid reaction rate, high yield, environmental friendliness, and low energy usage. Firstly, 3.49 gm of CuSO_4_·5H_2_O, 3.54 gm of ascorbic acid, 3.9 gm of NaOH, and 1.2 gm of starch, were separately dissolved in 100 ml of DI water. The preparation starts with robust stirring of 100 ml of Cu solution with 120 ml of starch solution for half an hour at 1500 rpm. Then, 50 ml of ascorbic acid solution was added to the prepared solution with continuous stirring for another half an hour. After that, 30 ml of NaOH solution was added drop by drop to the prepared solution with continuous stirring and heating at 80 °C for 2 h (the pH of the solution = 7). The color of the solution turned from yellow to ochre which indicates CuNPs formation. The solution was kept overnight to cool down then, precipitated by centrifugation at 9000 rpm at 20 °C for 20 min. The precipitate was washed carefully to remove the excess starch (3 times with DI water followed by 3 times with ethanol). Finally, the nanoparticles obtained were kept overnight on filter paper to dry to remove the remaining ethanol then stored in a glass vial for further analysis.

### Liposomal preparation and CuNPs loading

SLP was prepared by the thin film hydration method. It is the simplest method for SLP formation because it has a low encapsulation efficiency, but we treated it by controlling the size of the SLP, the temperature during preparation, and choosing the main concentration for SL and cholesterol^25^. 0.9 gm of SL and 0.1 gm of cholesterol dissolved in chloroform in a round bottom beaker, and using a rotary evaporator to dematerialize all chloroform to gain a thin film under vacuum at a temperature of 52 °C, maintained under vacuum conditions overnight to remove all traces of the detergent^26^. Then, 0.016 gm of CuNPs were dissolved in 40 ml of saline by stirring for 10 min. Then, add 30 ml of CuNPs solution to the bottom flask to dissolve the thin film by vortexing followed by sonication for 60 min to ensure the total solubility of the thin film in CuNPs solution. The obtained CuNPs solution and Cu/SLP dispersion were stored at 3–8 °C.

### Determination size and zeta potential

A dynamic light scattering (DLS) Zetasizer Nanoseries (Malvern Instruments, UK) analyzer was used to find out the particle size and zeta potential of CuNPs, SLP, and Cu/SLP. This analyzer gives information on particle size, zeta potential, concentration, and molecular weight. The samples were diluted 20 times before measurement. Set the detection angle at 90°, the temperature at 25 °C, and the refractive index. Using a helium–neon laser beam and a suitable cuvette for sample analysis^26^.

### Transmission electron microscopy

The morphology of the prepared samples, CuNPs, SLP, and Cu/SLP, was looked at with a JEM-2100 HR TEM [200 kV; JEOL, Japan; National Research Center, Cairo, Egypt]. The samples were prepared by placing a drop of phosphor-tungsten acid (a transmittance-negative stain) on carbon-coated copper and leaving it to air-dry before imaging.

### Molecular structure characterization

FTIR spectroscopy (4600 type A; National Research Center, Cairo, Egypt) was used to look at the chemical structures of CuNPs, SLP, and Cu/SLP. KBr sandwiches were pelleted perfectly, and all samples were prepared by mixing them separately. Spectra were acquired in the range of 4000–400 cm^−1^ with a resolution of 4 cm^−1^. Background reviews were acquired and abated from the diapason of the sample^26^.

### Standard curve for copper nanoparticles

The CuNPs concentration is 0.0349 mg/ml. Different concentrations of CuNPs are prepared, and their absorbance at wavelength 570 nm is measured using UV spectrophotometry, with the JENWAY 6405 UV/Vis Spectrophotometer (American Laboratory Trading, Inc., San Diego, USA). The standard curve of CuNPs is made as a relation between concentration (μg/ml) on the X-axis and absorbance (a.u.)on the Y-axis.

### Drug loading efficiency (DLE)

About 2 ml of Cu/SLP was diluted in 2 ml of saline solution (pH = 7.2) using ultracentrifugation at 12,000 rpm for 15 min at 4 °C to separate the free CuNPs from the ones that were encapsulated. The free CuNPs were found in the supernatant. Then take 1 ml from the supernatant, which contains the free CuNPs, to measure its absorbance by using a fluorimeter apparatus. From the CuNPs standard curve, the corresponding concentration is measured. By using the solution dilution calculator to determine the residual concentration (C_1_V_1_ = C_2_V_2_), the CuNPs loading efficiency (*DLE*) of Cu/SLP is calculated using Eq. (1)^27^.

1 $$DLE\;(100\% ) = \frac{Total\;drug - Free\;\,drug}{{Total\;drug}}\; \times \;100$$

### Drug release

A dialysis bag made from regenerated cellulose (RC) with glycerol as a protector from embrittlement. It's suitable for a pH range of 2–12 and a temperature of 4–60 °C with a diameter of 21 mm and a length of 5 m with a molecular weight cutoff of 5 KDa (about 5 cm of the dialysis tubing bag used in our experiment). The tube was washed in distilled water several times and filled with 3 ml of Cu/SLP followed by closing and descending the bag with buckles, then placed in a beaker containing 50 ml of NaCl saline or until the bag was covered with saline. The beaker was placed on low shift throughout the trial at room temperature. Samples were taken every hour for 6 h. After each sample, the same volume of fresh saline is added. The corresponding absorbance is measured for each sample using the spectrophotometer outfit. Eventually, the medicine release curve is colluded with time and absorbance^28^.

### In vitro cytotoxicity

For both the CuNPs and the Cu/SLP samples, the cytotoxicity test was done on a normal cell line (*Vero*) and a human breast cancer cell line (*Mcf-7*) from the Egyptian Holding Company for Biopharmaceuticals and Vaccines Production (VACSERA) in Cairo, Egypt. *Mcf-7* cells were seeded at a density of the order of 1 × 10^5^ cells/ml (100 μl / well) that was dispensed into a 96-well tissue culture plate and incubated at 37 °C for 24 h to develop a complete monolayer sheet. The two-fold dilutions of tested samples were made in RPMI medium with 2% serum^28^. 0.1 ml of every dilution was tested in different wells (leaving 3 wells as controls receiving only maintenance medium)^26^. Then the plate was incubated at 37 °C and examined. MTT solution was prepared (5 mg/ml in PBS). 20 μl of MTT solution was added to every well and placed on a shaking table (150 rpm for 5 min) to blend MTT with the media^26^. Incubate (37 °C, 5% CO_2_) for 4 h to permit the MTT to be metabolized. Soak the plate in paper napkins to get rid of the residue. Re-suspend formazan in 200 μl DMSO and place it on a shaking table (150 rpm for 5 min). The optical density at 560 nm and the background at 620 nm are recorded^26,29^.

### Statistical analysis of data

All data were expressed as the mean ± standard deviation (SD). The statistical analysis was conducted using Microsoft Excel 2010, and Origin 2018, with a p-value less than 0.05 for all data to be considered statistically significant.

## Results

The prepared CuNPs and Cu/SLP are collected, and further analyses are performed to ensure the proper preparation and their cytotoxic effect as shown below.

### Determination size and zeta potential

**Figure 1:**
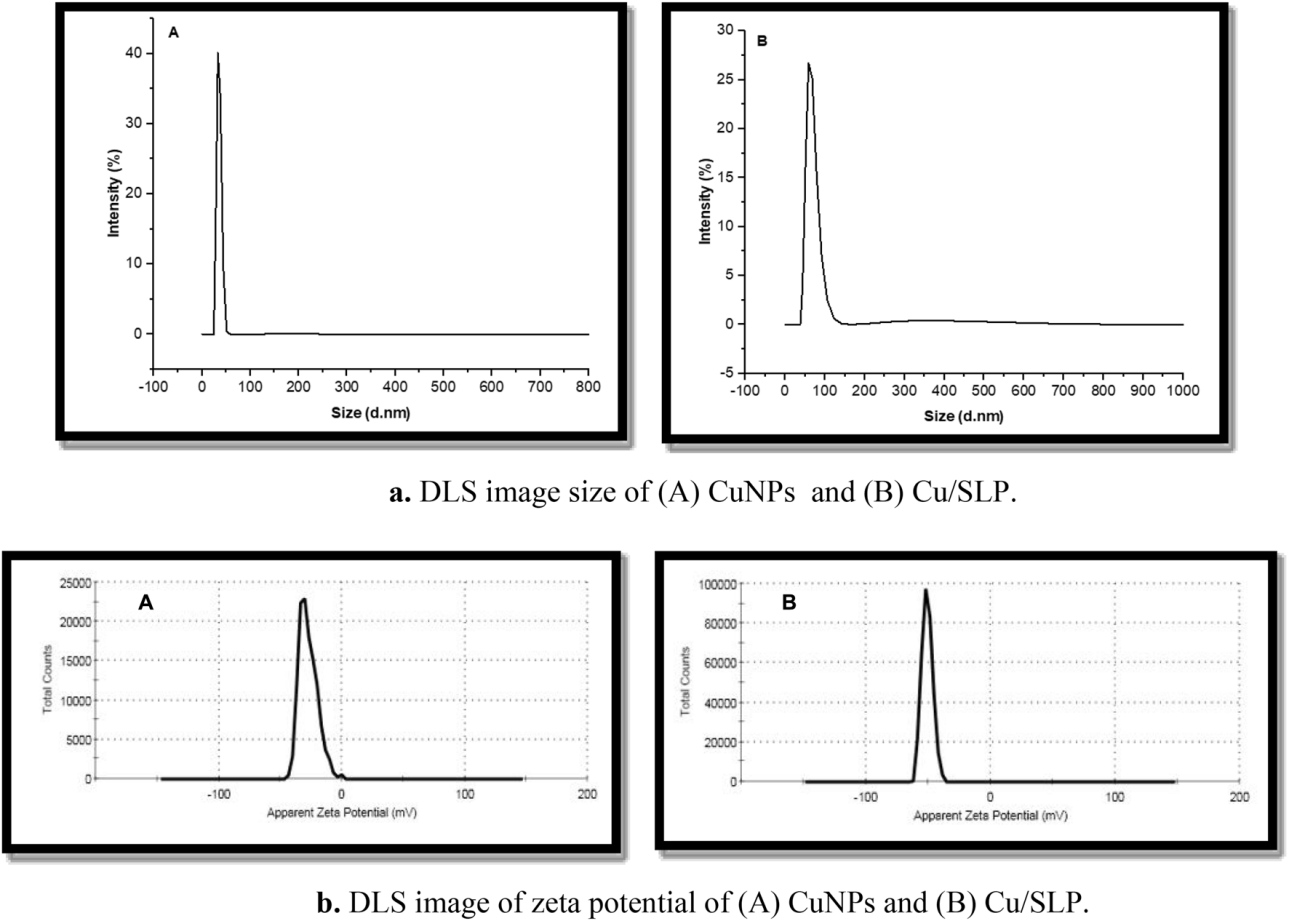
(**a**) DLS image size of (A) CuNPs and (B) Cu/SLP. (**b**) DLS image of zeta potential of (A) CuNPs and (B) Cu/SLP.

The size and zeta potential of CuNPs were measured to be 34.88 ± 4.53 nm with − 27.3 ± 7.23 mV, respectively, while the size and zeta potential of the encapsulated Cu/SLP were measured to be 81.59 ± 14.93 nm with − 50.7 ± 4.34 mV, respectively, as shown in Fig. 1a,b.

### Transmission electron microscope (TEM)

**Figure 2:**
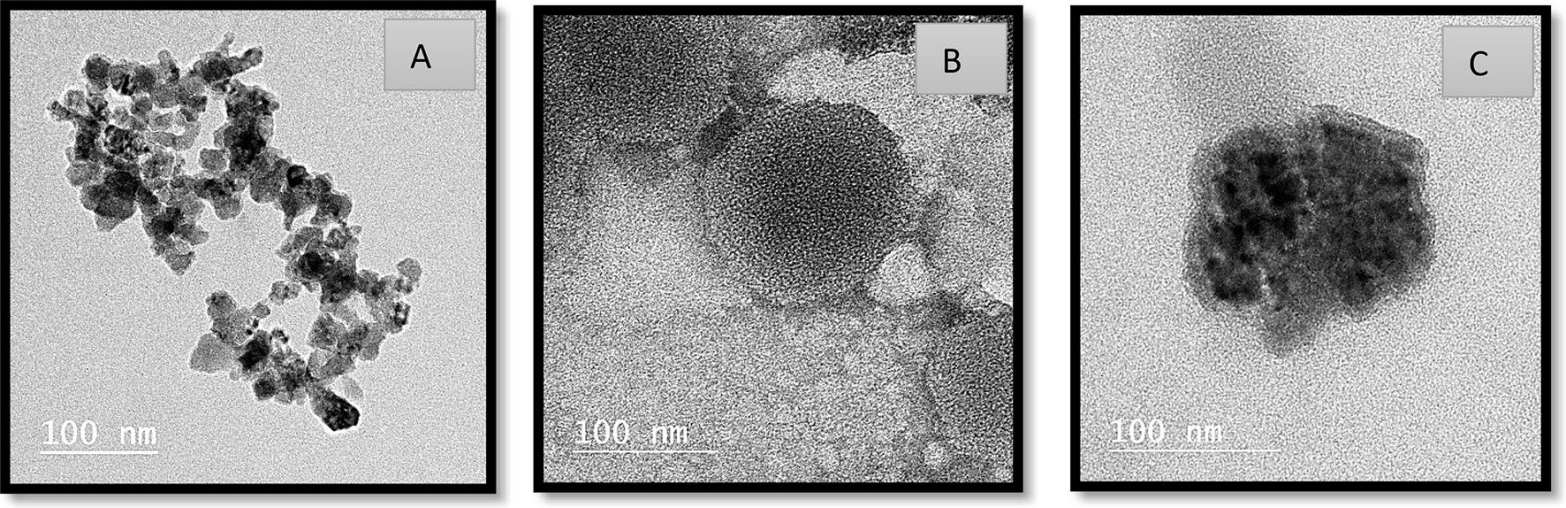
TEM images of (**A**) CuNPs, (**B**) SLP, and (**C**) CuNPs/SLP.

The morphology of both samples was further confirmed by TEM. This technique can highlight structural changes in any material. The samples were prepared by placing a drop from each sample on carbon-coated copper placed on filter paper, then placing a drop of transmittance-negative stain on it and leaving it to air-dry before imaging. As shown in Fig. 2, TEM verified the small size of CuNPs around 30 nm (Fig. 2A), and the presence of SLP in a round shape with multi-lamellar (Fig. 2B), while the Cu/SLP sample was round, dark in color because of CuNPs, and had a round shape with a diameter range less than 100 nm (Fig. 2C).

### Synthesis and characterization of SLP, CuNPs, and Cu/SLP

**Figure 3:**
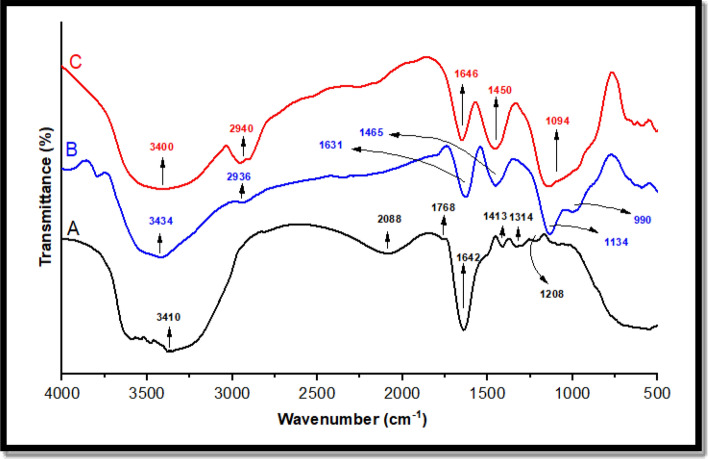
The Fourier transform infrared spectra of (**A**) SLP, (**B**) CuNPs, and (**C**) Cu/SLP.

The initial characterization of samples was performed using FTIR spectra, as shown in Fig. 3. Figure 3A–C represent the spectra of SLP, CuNPs, and CuNPs/SLP, respectively.

### Standard curve and drug loading efficiency

**Figure 4:**
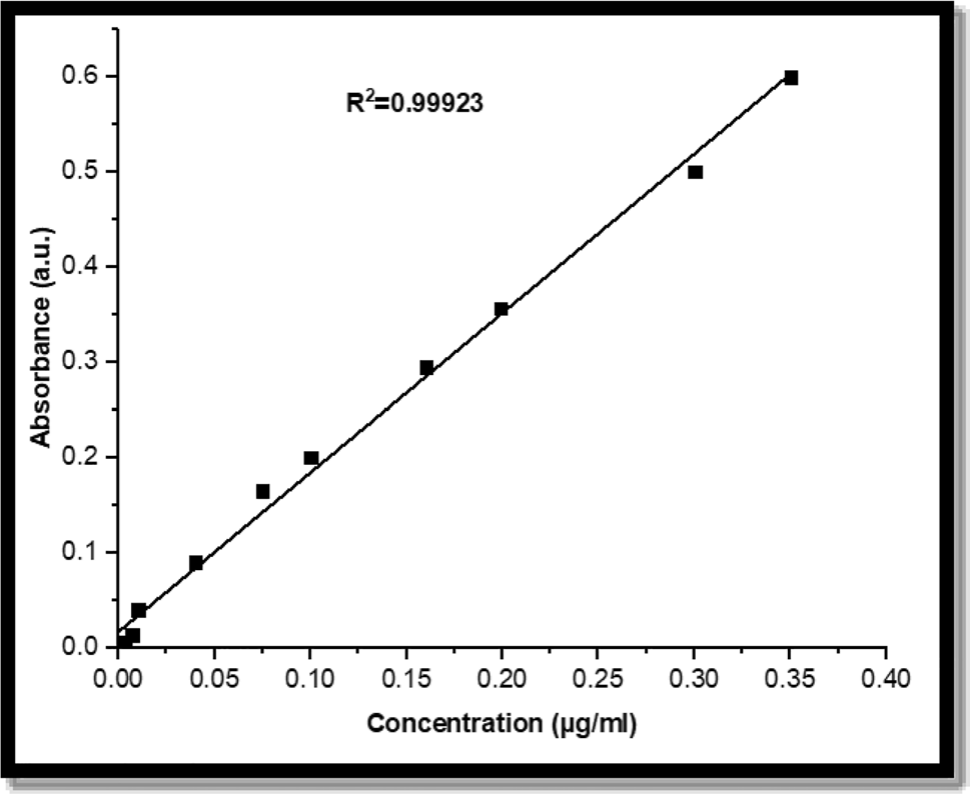
The standard curve for the CuNPs.

The absorbance spectrum was measured by the spectrophotometer and then plotted as a function of CuNPs concentrations, as shown in Fig. 4.

This curve will be used to calculate and measure the drug loading efficiency (DLE) of CuNPs.

### Drug release by dialysis bag

**Figure 5:**
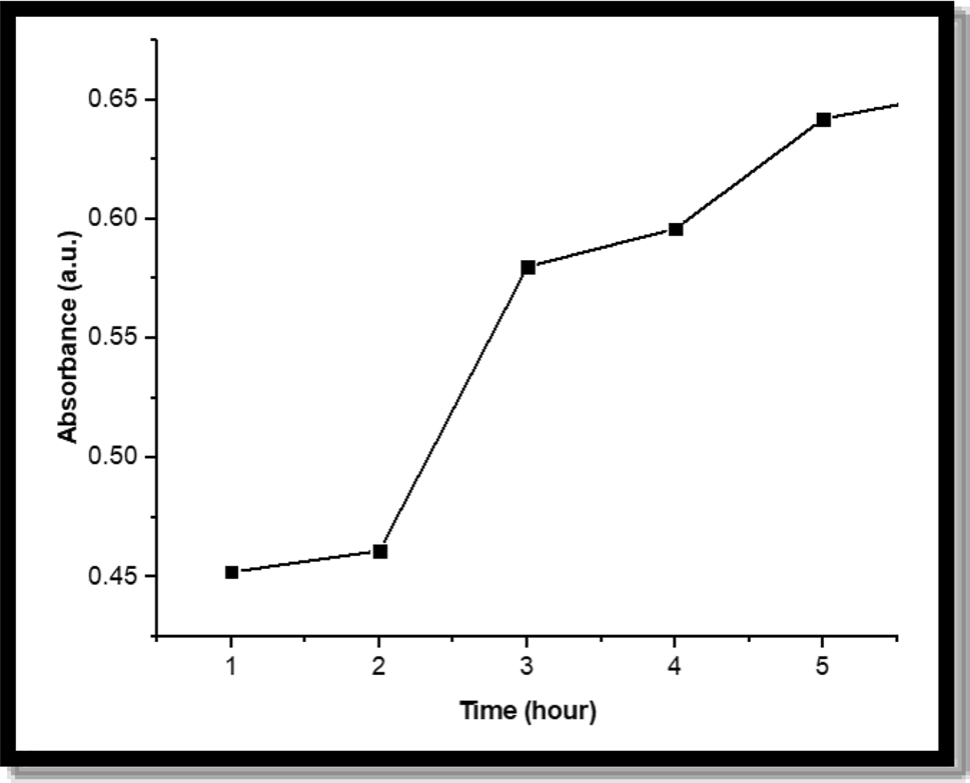
The release profile of CuNPs by dialysis bag.

The encapsulated CuNPs release was measured by measuring the absorbance of the dialysate as a function of time. As shown in Fig. 5, the plotted curve was the time in hours (h) on the X-axis and the absorbance (a.u.) on the Y-axis to get the release profile of CuNPs.

### Cell line cytotoxicity

**Figure 6:**
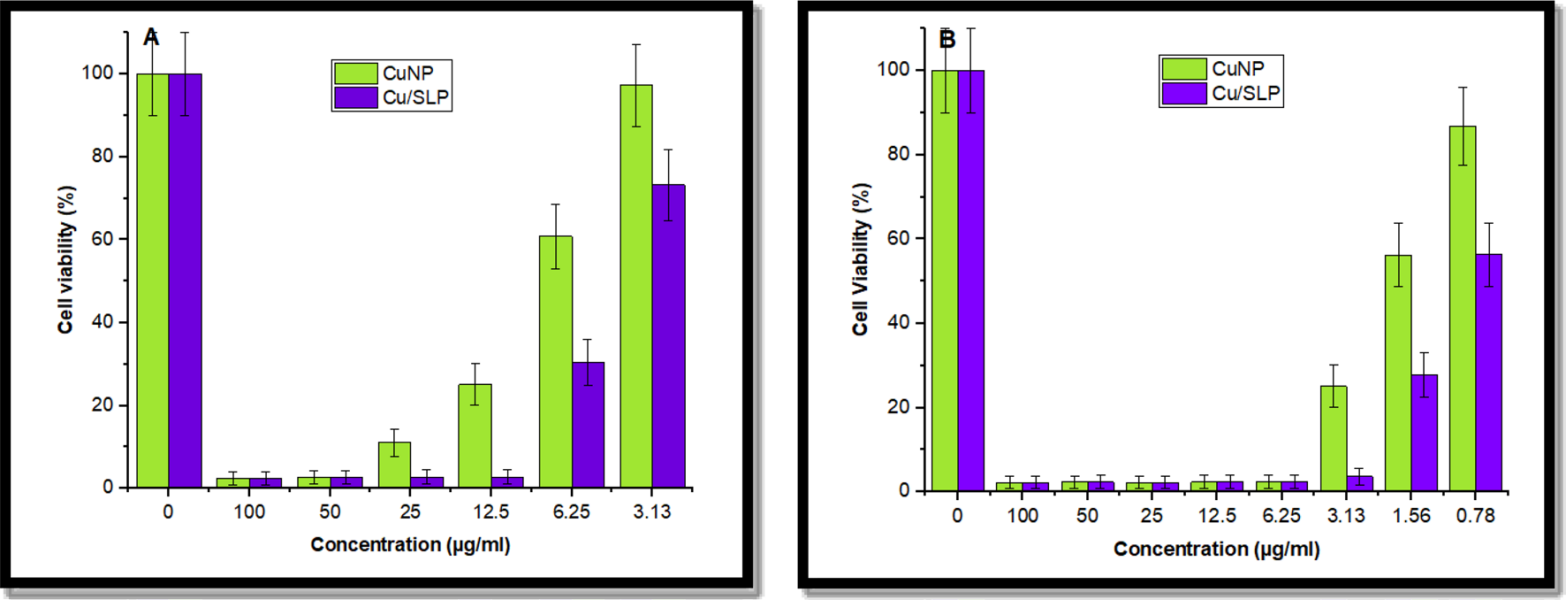
Cell viability of (**A**) *Vero* as a normal cell exposed to 100, 50, 25, 12.5, 6.25, and 3.125 μg/ml, (**B**) *Mcf-7* cells exposed to 100, 50, 25, 12.5, 6.25, 3.125, 1.56, and 0.78 μg/ml, of CuNP (green line) and Cu/SLP (violet line) on all.

AMTT assay was performed to evaluate the biocompatibility and toxicity due to the effect of chemotherapeutic agents on living cell lines. Here, CuNPs are used as a controlled drug compared to Cu/SLP as a drug model in both the normal cell line and the *Mcf-7* cell line. Table 1 illustrates the inhibitory effect of free CuNPs and Cu/SLP on a normal cell line (*Vero*) in different sample concentrations. While Table 2 represents the effect of CuNPs and Cu/SLP in different concentrations on *Mcf-7*. The cell viability of *Vero* cells as a normal cell line and *Mcf-7* exposed to different concentrations of CuNPs and Cu/SLP is measured. As shown in Fig. 6A, *Vero* as a normal cell is exposed to 100, 50, 25, 12.5, 6.25, and 3.125 μg/ml, while in Fig. 6B, *Mcf-7* cells are exposed to 100, 50, 25, 12.5, 6.25, 3.125, 1.56, and 0.78 μg/ml, of CuNP (green line) and Cu/SLP (violet line) on all.

**Table 1:** Chemo-sensitivity testing in a normal cell line (*Vero*) using an MTT assay. This table shows the results expressed as IC_50_, i.e., the concentration of cytotoxic drug that reduces cell viability by 50% relative to the control.

ID	µg/ml	O.D			Mean O.D	± SE	Viability %	Toxicity %	IC_50_ ± SD
*Vero*	–	0.74	0.74	0.73	0.74	0.00	100.00	0.00	µg/ml
CuNPs	100.00	0.02	0.02	0.02	0.02	0.00	2.40	97.60	8.72 ± 0.04
	50.00	0.02	0.02	0.02	0.02	0.00	2.58	97.42	
	25.00	0.08	0.08	0.09	0.08	0.00	11.10	88.90	
	12.50	0.18	0.19	0.18	0.18	0.00	25.09	74.91	
	6.25	0.46	0.44	0.45	0.45	0.01	60.78	39.22	
	3.13	0.72	0.72	0.71	0.72	0.00	97.24	2.76	
Cu/SLP	100.00	0.02	0.02	0.02	0.02	0.00	2.49	97.51	5.44 ± 0.09
	50.00	0.02	0.02	0.02	0.02	0.00	2.63	97.37	
	25.00	0.02	0.02	0.02	0.02	0.00	2.76	97.24	
	12.50	0.02	0.02	0.02	0.02	0.00	2.76	97.24	
	6.25	0.22	0.24	0.22	0.22	0.01	30.53	69.47	
	3.13	0.54	0.55	0.53	0.54	0.01	73.23	26.77

**Table 2:** Cu-sensitivity testing in the breast cell line (*Mcf-7*) using an MTT assay. This table shows the results expressed as IC_50_, i.e., the concentration of cytotoxic drug that reduces cell viability by 50% relative to the control.

ID	µg/ml	O.D			Mean O.D	± SE	Viability %	Toxicity %	IC_50_ ± SD
*Mcf-7*	–	0.79	0.80	0.79	0.80	0.00	100.00	0.00	µg/ml
CuNPs	100.00	0.02	0.02	0.02	0.02	0.00	2.26	97.74	2.05 ± 0.02
	50.00	0.02	0.02	0.02	0.02	0.00	2.31	97.69	
	25.00	0.02	0.02	0.02	0.02	0.00	2.26	97.74	
	12.50	0.02	0.02	0.02	0.02	0.00	2.35	97.65	
	6.25	0.02	0.02	0.02	0.02	0.00	2.43	97.57	
	3.13	0.22	0.18	0.20	0.20	0.01	25.12	74.88	
	1.56	0.44	0.46	0.44	0.45	0.01	56.14	43.86	
	0.78	0.69	0.69	0.69	0.69	0.00	86.79	13.21	
Cu/SLP	100.00	0.02	0.02	0.02	0.02	0.00	2.22	97.78	0.91 ± 0.08
	50.00	0.02	0.02	0.02	0.02	0.00	2.35	97.65	
	25.00	0.02	0.02	0.02	0.02	0.00	2.26	97.74	
	12.50	0.02	0.02	0.02	0.02	0.00	2.39	97.61	
	6.25	0.02	0.02	0.02	0.02	0.00	2.52	97.48	
	3.13	0.02	0.03	0.03	0.03	0.00	3.65	96.35	
	1.56	0.22	0.24	0.21	0.22	0.01	27.88	72.12	
	0.78	0.45	0.43	0.47	0.45	0.01	56.27	43.73

**Figure 7:**
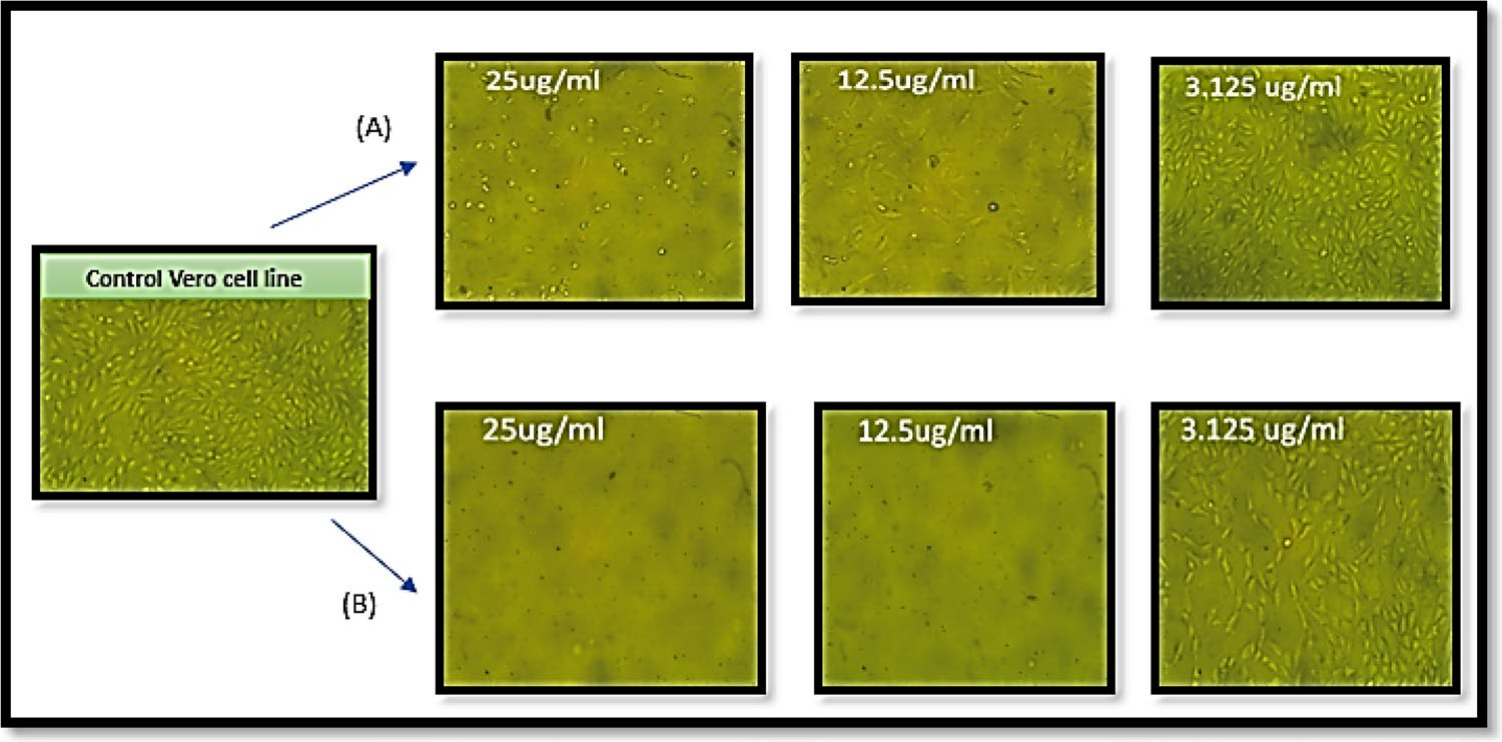
Morphology of *Vero* cells; a control *Vero* cell line; (**A**) the effect of free CuNPs with concentrations (25, 12.5, and 3.125 μg/ml) while (**B**) the effect of CuSLP with concentrations (25, 12.5, and 3.125 μg/ml).

**Figure 8:**
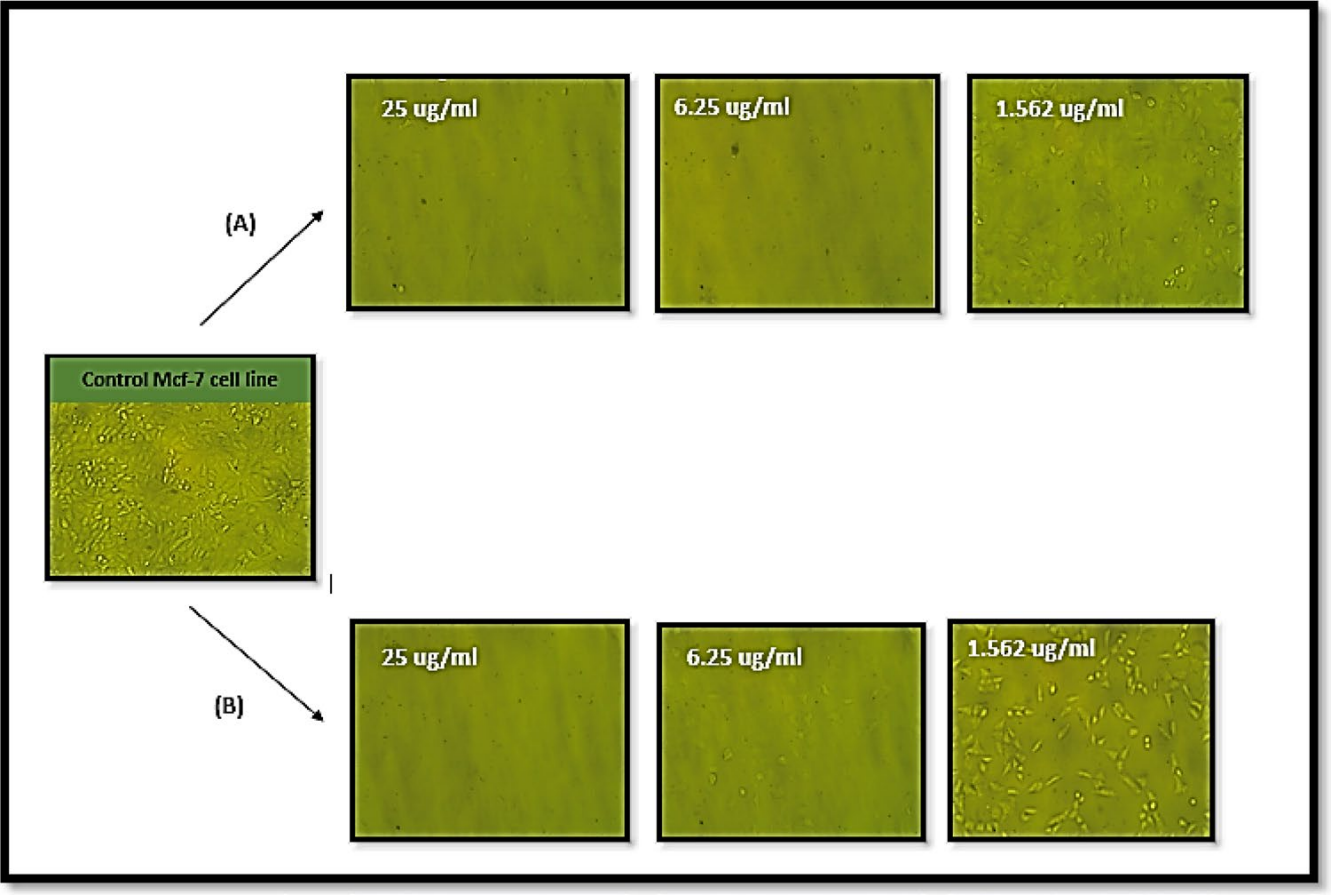
Morphology of *Mcf-7* cells; a control *Mcf-7* cell line, (**A**) the effect of free CuNPs with concentrations (25, 6.25, and 1.562 μg/ml), and (**B**) the effect of Cu/SLP with concentrations (25, 6.25, and 1.562 μg/ml).

The morphological changes caused by the action of CuNPs and Cu/SLP on *Vero* and *Mcf-7* cells are shown in Figs. 7 and 8, respectively.

Figure 7 describes the morphological changes of different sample concentrations on *Vero* cells with (25, 12.5, and 3.125 µg/ml) of (A) CuNPs and (B) Cu/SLP.

While Fig. 8 describes the morphological changes of different sample concentrations on *Mcf-7* cells with (25, 6.25, and 1.562 µg/ml) of (A) CuNPs and (B) Cu/SLP.

## Discussion

Copper nanoparticles (CuNPs) as a drug model for cancer treatment, either in their free form or encapsulated in Soy lecithin liposomes (SLP) from plant origin as a cheap source of lipids, are considered one of the potential drugs that can be used for cancer treatment. The synthesis of CuNPs is difficult owing to their high oxidation tendency. They are particularly sensitive to air and their oxide phases are thermodynamically more stable. The rapid oxidation rate of the CuNPs may limit their applicability. Therefore, the oxidation of CuNPs can be avoided, if their synthesis is performed in presence of CO or H_2_. However, treating these gases is time-consuming; thus, they can be avoided if possible. The rate of nanoparticles growth is affected by several factors, including metal ion concentration, reductant type, pH, and temperature. The process is based on low-temperature chemical reduction in an aqueous copper salt using ascorbic acid as a reducing agent, and starch was employed to control nanoparticles development and to protect them from oxidation and aggregation^30,31^. The synthesized CuNPs and Cu/SLP of size and zeta potential of 34.88 ± 4.53 nm and − 27.3 ± 7.23 mV for CuNPs and 81.59 ± 14.93 nm and − 50.7 ± 4.34 mV for the encapsulated Cu/SLP indicate the proper sample size and stability. The expansion of carriers with the proper charge and size plays an important role in drug delivery. The smaller size of NP improves the intracellular concentration, and accumulation of the drug in tumors^32^. As shown in Fig. 1, there is an increase in particle size because of the attractive force between Cu and SLP. The Cu/NPs in nanosized (≤ 100 nm) particles help to improve medical efficacy, by allowing the drug to move freely in the circulation of the human body and for a long time after release^33–35^. These structures can penetrate the tissue system, improve and facilitate the uptake of the drug by cells, permit efficient drug delivery, and ensure action at the targeted location. Also, this small size helps in the uptake of nanostructures by tumor cells much higher than that of large particle sizes which helps in reducing the side effects of the drug on healthy tissues^36^. The stability of Cu/SLP was measured by the value of the zeta potential. As shown in Fig. 1b, the potential increases from − 27.3 to − 50.7 mV because of the interaction between Cu and SLP^37^. That increase in negative charge not only helps to improve the physical stability of the sample (prevent sample aggregation), but also significantly reduces endocytosis compared to positive charge^38^. The TEM images showed that both CuNPs and Cu/SLP were spherical in morphology without any aggregation which correlated with DLS results, and the liposomal system possessed a size range that fell into the therapeutic-potential range^39^.

FTIR can also tell if there are functional groups on the surface of the nanoparticle and if there are proteins, carbonyl groups, and CH bonds in amines, which are the source of biosynthesis. According to wavenumber (cm^−1^), the band area values were divided into 3 linear baselines from 4000 to 2500 cm^−1^, from 2500 to 1500 cm^−1^, and finally the fingerprint lines of the samples from 1500 to 300 cm^−1^^40^. As shown in Fig. 3, in the spectra of SLP, CuNPs, and Cu/SLP. FTIR spectrum of SLP (Fig. 3A) showed a characteristic broad band centered at approximately 3410 cm^−1^ that was assigned to the stretching vibration of the O–H group. The bands at approximately 2088 cm^−1^ are related to the isothiocyanates vibration of (N=C=S), whereas the band at 1768 cm^−1^ is attributed to the stretching vibration of (C=O). At approximately 1642 cm^−1^, which is assigned to the C=C stretching vibration^41,42^. The small bands at 1413 and 1314 cm^−1^ are related to scissoring CH_2_ vibration and abroad medium band of C–O vibration, respectively^43^. The band at 1208 cm^−1^ is a characteristic phosphate group vibrational band related to PO_2_ as an antisymmetric stretching mode^44,45^. The spectrum of CuNPs (Fig. 3B) illustrates a broad band at approximately 3434 cm^−1^, which is referred to the N–H stretching vibration mode, while at approximately 2936 and 990 cm^−1^ the bands were caused by a strong stretching C–H vibration^46^. The band at 1631 cm^−1^ was due to C=C, a strong stretching vibration, and the band at 1465 cm^−1^ was due to the bending vibration of the C–H bond. The band at approximately 1134 cm^−1^ is related to the C–O symmetric vibration^47,48^. In the Cu/SLP spectrum (Fig. 3C), the broadband at 3400 cm^−1^ may be due to the interference between the CuNPs and SLP. At 1646 cm^−1^, the intensity of the band as compared to the SLP spectrum decreased reflecting the type of interaction that occurred between CuNPs and SLP. The other bands which are present at 2940 cm^−1^, strong stretching C–H vibration, 1450 cm^−1^ with strong stretching C=C vibration, and 1094 cm^−1^ with strong alcohol stretching C–O vibration are related to the presence of CuNPs^49^. Any chemical shift in the bands of Cu/SLP when compared to SLP and CuNPs spectrums suggests that encapsulation occurred without any changes to the chemical bonds of the nanoliposome^50^. The drug loading efficiency (DLE) of the prepared Cu/SLP was measured using Fig. 4 and Eq. (1) to be 78.9%. According to this result, as drug encapsulation in SLP increased, it improved the pharmacokinetics of the drug (here, CuNPs as a drug). Where CuNP is a hydrophilic drug, it can easily dissolve in the external aqueous phase during the preparation of liposomes, which led CuNP to defuse into the core of SLP, ending with an increased EE%^51^. Also, the DLE does not depend only on drug solubility but also depends on the size of the liposome, drug interaction with the liposome, thermal conductivity of the drug, and the presence of functional groups on either Cu or SLP. All these factors help to increase DLE %^52,53^. Drug loading capacity is an important factor that shows us the stability degree of our sample and the stability of the drug carrier system. From our results, the Cu/SLP size, the thermal conductivity of Cu, and the functional groups on the surfaces of both CuNPs and SLP help to improve the DLE of the sample to approximately 80%^54^.

The drug release profile of Cu/SLP, as shown in Fig. 5 was very slow for 4 h and continued to increase until the end of the experiment. From the standard curve, it is found that after 2 h only 27% of the total Cu concentration in the bag was released, while after 5 h, about 41% of the total Cu concentration was released. It can be estimated that after approximately 21 h, about 99% of Cu will release from the SLP. This result indicates a slow release which is attributed to the electrostatic attraction between Cu and SLP^55^. This attraction supports Cu stability inside the SLP and leads to an increase in the lifetime of Cu/SLP in blood circulation. This helps to get to the main point of our research: decreasing the side effects of Cu on the healthy organ by loading it on SL from plant origin^56^. Also, the low size of Cu/SLP, stability of zeta potential, storage conditions, and material of high quality all precipitate in the release profile of the sample and prevent drug leakage^57^. A MTT assay was carried out to evaluate the biocompatibility of Cu/SLP. The basic premise of the assay is that mitochondrial dehydrogenases in live cells break the tetrazolium ring of the membrane-permeable MTT dye, creating purple-colored insoluble formazan crystals that, when dissolved in acidified isopropanol or DMSO, have the highest absorbance at 570 nm^58^. As less tetrazolium cleavage occurred when greater cell death occurred, the magnitude of the drop at λ = 570 nm served as a reflection of cytotoxicity. CuNP-treated cells' growth inhibition was expressed as a percentage of the inhibitory concentration (IC) compared to the untreated control cells. The results are expressed as the percentage of cell proliferation, calculated as follows:

Viability (%) = (mean OD treated − mean OD background)/(mean OD untreated cultured) × 100^59^.

The cell viability assay, which is described in the histograms ± SD of all analyses, is shown in Fig. 6, and can be used to determine the effect of Cu/SLP on *Mcf-7* and to optimize the cell culture conditions^60^.

As shown in Table 1 and Fig. 6A, a normal cell line (*Vero*) was exposed to different concentrations of 100, 50, 25, 12.5, 6.25, and 3.125 µg/ml of both CuNPs and Cu/SLP separately. Comparing the results of cell viability of both found that in CuNPs, the cell viability decreases from low to high concentration, but in Cu/SLP, the cell viability is stable as the concentration increases. This means that as the concentration increases, the cell’s health is stable in response to different sample concentrations. IC_50_ ± SD of both CuNPs and Cu/SLP was 8.72 ± 0.04 and 5.44 ± 0.09 µg/ml, respectively. This effect can be attributed to the efficiency of the encapsulation of Cu/SLP, which controls the drug release during cell incubation^61^. In 2020, research investigated the cytotoxicity of Cu/CuO NPs in normal *lung and lung cancer cell lines*. IC_50_ was 201.26 µgm/ml in a normal lung cell line while IC_50_ was 209.7 µg/ml in a lung cancer cell line^47^. Although the effect of SLP on cancer cells has not been investigated, different investigations have found that ((Le, et al. 2021) the blank PEGylated liposomes had a minor effect on the viability of *Mcf-7 cell lines*, with more than 80% of the cells surviving at all concentrations examined (0, 50, 100, 250, and 500 g/ml), and *MCF-7* images clearly demonstrated that when treated with blank PEGylated liposomes at high concentrations up to 500 g/ml, the majority of the cells retained their unique morphology. These findings demonstrate that PEGylated liposomes are biocompatible carriers with no cytotoxicity^62^. In addition, Nguyen et al. (2017) prepared SLP and platexil loaded with SLP using the thin film method and investigated the effect of SLP on *Mcf-7.* SLP showed no discernible cytotoxicity in the *Mcf-7 cell line.* Almost 100% of cells remained alive after 2 days in 500 g/mL samples, demonstrating that the lips are biocompatible^63^.

As shown in Table 2 and Fig. 6B, the *Mcf-7* cell line was exposed to different concentrations of 100, 50, 25, 12.5, 6.25, 3.125, 1.526, and 0.781 µg/ml of both CuNPs and Cu/SLP separately. By comparing the concentration of the drug that needs to kill 50% of *Mcf-7*, it is lower in Cu/SLP in comparison to CuNPs (IC_50_ ± SD is 0.91 ± 0.08 and 2.05 ± 0.02 µg/ml respectively). These results confirm the enhanced cytotoxicity of Cu/SLP in comparison to free CuNPs. In comparison with other drugs loaded by liposomes used for cytotoxicity of the *Mcf-7 cell line*, in 2023, the IC_50_ ± SD of Dox/SLP was 4.81 ± 0.07 µg/ml^26^, also, in 2022, the IC_50_ ± SD of curcumin-loaded-liposomes was 11.5 ± 1.1 μg/ml^64^, in 2021, the IC_50_ ± SD of docetaxel loaded liposome was 3.56 ± 0.27 µg/ml^65^. Cu/SLP was found to be the best choice for killing 50% of *Mcf-7* cells. The shape and small size of SLP play an important role in the biological effect, especially cellular uptake and biodistribution^66^. Cu/SLP travels through the circulation in a safe manner from the reticuloendothelial system until it reaches the tumor site, where it releases Cu in the tumor cell, prompting apoptosis through the mitochondrial pathway, by increasing the production of reactive oxygen species and oxidative stress, which finally leads to DNA damage and cell death^67^. Also, as the time of the release of Cu from SLP increases, it helps to increase the half-life of Cu in blood circulation, which helps us reach the main point of our research: using the minimum concentration of the drug to kill cancer cells by loading the drug on a transporter from plant origin to deliver it with the minimum side effects to the rest of the body organs. Finally, from an in vitro study on the *Mcf-7* cell line, it is concluded that as the concentration of Cu/SLP increases, there is low sample toxicity in comparison to free CuNPs. It can be returned to the correlation of binding forces between Cu and SLP. Where SLP acts as a transporter for Cu, protecting Cu from RES, SLP helps to deliver the almost full dose to the tumor site and decreases the side effects of Cu on healthy tissue. All these factors contribute to the lower IC_50_ of Cu/SLP^68^. The morphological changes caused by the action of CuNPs and Cu/SLP on *Vero* and *Mcf-7* cells are shown in Figs. 7 and 8, respectively. As shown, there is a clear difference between the effects of CuNPs and Cu/SLP on cells.

## Conclusion

In this study, the thin film hydration method was used to successfully synthesize SLP from soy lecithin, which comes from plants. This was done to deliver CuNPs. The characterization of the sample proves the success of encapsulation and high stability of the sample, as indicated from DLS results with a mean size range of 81.59 ± 14.93 nm and a zeta potential of − 50.7 ± 4.34 mV, which is consistent with the measured size by TEM. The prepared sample has a high drug loading efficiency of approximately 80% and CuNPs were released from Cu/SLP in a controlled manner. In addition, the IC_50_ ± SD for Cu/SLP (0.91 ± 0.08 µg/ml) is smaller than that of free CuNPs (2.05 ± 0.02 µg/ml). SLP from plant origin is considered one of the potential candidates used in drug delivery as in doxorubicin from an economic point of view because SL is much cheaper than any other lipid source in addition to its electrical and thermal properties. Thus, Cu/SLP is considered a perfect choice for cancer treatment because it has a great effect on cancer cells at much lower concentrations which will lead to a decrease in the side effects of chemotherapy on healthy tissues.

## Data Availability

All data are available from the corresponding author upon request.

## Abbreviations

CuNPs: Copper nanoparticles
Cu/SLP: Copper nanoparticles loaded by soy lecithin liposome
DI: Deionized water
DLE: Drug loading efficiency
EE%: Encapsulation efficiency
FTIR: Fourier transforms infrared
MW: Molecular weight
RES: Reticuloendothelial system
TEM: Transmission electron microscopy
SLP: Soy Lecithin liposome
